# A genome-wide deletion map in 125,730 individuals for novel rare disease gene and variant discovery

**DOI:** 10.64898/2026.05.13.26352722

**Published:** 2026-05-15

**Authors:** Anthony McGuigan, Alistair T. Pagnamenta, Laura E. Covill, Jacob Samson, Carme Camps, Yuyang Chen, Tanishi Moitra, Kartik Chundru, Emily O’Heir, Kirsten Allan, Gavin Arno, Alexander Broomfield, Martin Delatycki, Siying Lin, Michel Michaelides, Rocio Rius, Tony Roscioli, Cas Simons, Andrew Webster, Susan M. White, Louise Wilson, Stephan J. Sanders, Anne O’Donnell-Luria, Jamie M. Ellingford, Jenny C. Taylor, Nicola Whiffin

## Abstract

Structural variants (SVs) can disrupt gene function and contribute to pathogenesis of rare disorders. Here, we created a genome-wide knockout dataset across 125,730 individuals with genome sequencing data in the UK’s National Genomic Research Library by leveraging the distinct read-depth signal associated with homozygous deletions. We curated 535,699 rare high-confidence homozygous deletion SVs, of which 48,735 were rare. These deletions collectively covered 213Mb or 6.92% of the human genome (4.58% of autosomal sequence), revealing substantial tolerance to complete sequence loss. From a subset of 58,022 individuals with rare disease, we identified 295 individuals with likely diagnostic homozygous deletions impacting protein-coding regions of known disease genes. A further 32 individuals had candidate non-coding SVs in or near to known disease genes, 19/32 (59.37%) of which disrupted 5’-UTR/promoter regions, revealing promoter deletion as an underappreciated cause of rare disorders. Finally, we identify 43 genes with no known rare-disease association but with exonic homozygous deletions in two or more individuals with consistent phenotypes. We describe in detail *PDC* (phosducin) in Leber Congenital Amaurosis, *GCG* (glucagon) for a syndromic neurodevelopmental disorder with gastrointestinal involvement, and *ENTPD3* for intellectual disability with autism, as candidate novel disease-associated genes. Overall, we create a genome-wide map of homozygous deletions and demonstrate the power of this dataset for rare disease diagnosis and novel disease-gene discovery.

## Full Text Availability

The license terms selected by the author(s) for this preprint version do not permit archiving in PMC. The full text is available from the preprint server.

